# Poor Executive Functions among Children with Moderate-into-Severe Asthma: Evidence from WCST Performance

**DOI:** 10.3389/fpsyg.2017.00793

**Published:** 2017-05-16

**Authors:** Haitham Taha

**Affiliations:** ^1^The Cognitive Lab for Learning and Reading Research, Sakhnin CollegeSakhnin, Israel; ^2^Safra Brain Research Center for Learning Disabilities, University of HaifaHaifa, Israel

**Keywords:** asthma, learning disabilities, executive functions, WCST, learning skills

## Abstract

Executive functions (EFs) measures of 27 asthmatic children, with general learning difficulties, were tested by using the Wisconsin card sorting test (WCST), and were compared to the performances of 30 non-asthmatic children with general learning difficulties. The results revealed that the asthmatic group has poor performance through all the WCST psychometric parameters and especially the perseverative errors one. The results were discussed in light of the postulation that poor EFs could be associated with the learning difficulties of asthmatic children. Neurophysiological framework has been suggested to explain the etiology of poor EFs among children with moderate into severe asthma.

## Introduction

Asthma is one of the common inflammatory diseases that could negatively affect the quality of children’s life (Lavoie et al., 2005). As a disease, asthma is characterized by wheezing, breathlessness, and chest tightness while the prevalence of asthma is about 7–8% in childhood and adolescence (National Center for Health Statistics [NCHS], 2012; Scottish Intercollegiate Guidelines Network [SIGN], 2013). Etiologically, Asthma is thought to be caused by a combination of genetic and environmental factors (Martinez, 2007; Dietert, 2011).

Recent developmental studies had consistently reported about the relationship between asthma and other childhood developmental disorders. For example, data from the “1988 US National Health Interview Survey on Child Health,” a nationally representative cross-sectional survey, was used to determine national estimates of school outcomes (Fowler et al., 1992). According to this survey, a modestly increased risk of academic problems among children with asthma compared with typical children was suggested. In another recent survey that was conducted by Blackman and Gurka (2007), a sample of 102,353 randomly selected children ages 0–17 years was tested to explore the prevalence of developmental and behavioral comorbidities with asthma. According to this survey, Children with asthma have higher rates of attention deficit hyperactivity disorder (ADHD), diagnoses of depression, behavioral disorders, and learning disabilities. Accordingly, children with asthma may encounter in higher risks for emotional and cognitive disorders than typical children (Blackman and Gurka, 2007).

In spite of the fact that ADHD is considered as the most reported disorder which has high comorbidity with asthma, this relationship between Asthma and ADHD is not clear (Chen et al., 2013). Different postulations have been suggested in aim to explain such comorbidity. For example, it has been suggested that cortisol changes in children with mild and moderate asthma play a modest role in the neuropsychological functions which affect self-regulation (Annett et al., 2005).

Another common explanation behind this comorbidity relates to the asthma medication itself and specifically the inhaled corticosteroids (ICS) due to the reporting of behavioral disturbances, developmental disabilities and psychosis in children using inhaled ICS (Bonala et al., 2003). Contrary to the later explanation, different studies do not prove these correlations (see for example; De Vries et al., 2008). Other explanations suggested that environmental factors is behind this comorbidity, such as parents’ smoking habits, low birth weight and low socioeconomic status that could contribute negatively to the child’s health and his cognitive development as well (Strachan, 2000; Goodwin, 2007). Genetic factors were also suggested within the risk factors that could contribute to the high comorbidity between Asthma and ADHD (Fasmer et al., 2011; Mogensen et al., 2011; Chen et al., 2013).

Attention deficit hyperactivity disorder, as behavioral disorder, is mainly considered as a disorder of executive functions, (EFs), (Barkley, 1997, 2001). In addition, poor EFs were associated with the learning difficulties and self-regulation problems among children with asthma that have ADHD also (Annett and Bender, 1994; Bender, 1995; Yuksel et al., 2008; Pelsser et al., 2009; Fasmer et al., 2011; Mogensen et al., 2011; Fryt et al., 2013).

Executive function is defined as an umbrella term for the management, regulation and control of the cognitive processing (Lezak, 2004, p. 611). In addition, cognitive flexibility, as a main component of EF, is thought to affect the quality of the cognitive processing control (Anderson, 1998). It is well evident that the quality of EF determines the effectiveness of the learning abilities and specially the learning of high cognitive demanding skills due to the need of highly efficient EF (Kehagia et al., 2010; Best et al., 2011). Accordingly, poor EF may be a main cognitive consequence of asthma (Annett and Bender, 1994; Yuksel et al., 2008; Pelsser et al., 2009; Mogensen et al., 2011; Fryt et al., 2013) or may be one of the poor cognitive consequences of such comorbidity with ADHD (Barkley, 2001). If EF difficulties are associated with asthma itself and not a consequence of comorbidity with ADHD, accordingly, Asthmatic children will encounter with difficulties in performing tasks that measuring EF. If this is the case, can EF difficulties be associated with learning difficulties among Asthmatic children?

Consistently, the aim of the current study is to investigate whether asthmatic children with general learning difficulties encounter with poor EF as a suggested source of their learning difficulties.

Different tasks were suggested for investigating the EF. For example the Wisconsin Card Sorting Test (WCST) is a common neuropsychological test that is widely being used for testing the ability to display flexibility in the face of changing schedules of reinforcement (Grant and Berg, 1948; Heaton et al., 1993).The level of the different errors during the performance in the WCST could be considered as main index of the EF. Accordingly, assessing the EF in the current study will be conducted by using the WCST and comparing the different levels of errors between two groups of children with learning difficulties, asthmatic and non-asthmatic children.

## Materials and Methods

### Participants

A total number of 27 children (17 boys and 11 girls) with persistent general learning difficulties and documented history of chronic moderate into severe asthma were selected to participate in the current study (mean age = 12.46 ± 0.43). The information about the medical history of the participants was gathered during the clinical intake with their parents. The WCST performance of this group of participants was compared to the performance of another 30 children with general learning difficulties (19 boys and 11 girls) without any historical evidence of having asthma in any period of their lifespan (mean age = 12.5 ± 0.45). All the data was obtained from data base of private clinic for learning disabilities diagnosis in north of Israel. All the participants attended the clinic for having a professional diagnosis in aim to determine the source of their learning difficulties.

The participants fell below the 15 percentile of their class according to their school data considering their academic achievements. Any sensory or emotional disturbances were reported. All the participants were right handed and from the middle socioeconomic class. For each participant, the parents were informed that the data from this test will be used for further research purposes and they were asked to give their approval for using their child data. The data that was used within the current research belongs to the children who had parents’ approval to participate the current study. In addition, the current study was conducted after the approval of the research ethics committee of the Sakhnin academic college for education in Israel.

### Material and Stimuli

Each participant was tested with the computerized WCST-64: Computer Version 2, Research Edition (WCST-64^TM^:CV2, Robert K. Heaton, Ph.D. and PAR Staff, Psychological Assessment Resources, 2003). For performing the test, each participant was tested individually and was seated at a distance of ∼70 cm in front of 21″ computer screen. The test takes approximately 15–20 min to carry out and generates a number of psychometric scores, including numbers, percentages, and percentiles of: total errors, perseverative errors, perseverative responses and non-perseverative errors.

The total errors parameter indexes EF in general, while the perseverative errors and perseverative responses are measurers of cognitive flexibility. It is important to justify that while the number of the perseverative errors and responses increases there is a reduced level of cognitive flexibility (Grant and Berg, 1948). In addition, the non-perseverative errors index mainly measures the controlling the attentional resources and self-mentoring.

While performing the WCST, four target cards are being presented to the subject on the computer display. Then, different cards start to be sequentially displayed one by one. During all this presentation, the subject is required to match each displayed card according to one of the four target cards shown in face. The subject is not being told how to sort but s∖he is required to deduce the sorting principle (by color, shape or number) according the test response (right or wrong). When the subject reveals the principle of sorting, s/he continues the sorting operation while maintaining a continuous recruitment of sustained attention and continuous concentration for not being confused. At some point, the testing tool replaces the sorting principle, and the test responses “wrong” to the sorting responses that the participant makes according to the former sorting principle, indicating that the sorting principle was changed. Once the participant notices that the sorting principle was changed, s/he needs to deduce the new one by examining different sort options in flexible way. Incorrect response and continued working under the previous sorting principle, in spite of the “wrong” warnings, are considered as perseverative responses that could lead to perseverative errors. As the number of the perseverative errors increases there is a reduced level of flexibility (Grant and Berg, 1948). It is important to note that the perseverative responses are not always revealing into perseverative errors and this is why the number of the perseverative errors is different from the perseverative and the non-perseverative responses.

The WCST is being widely used as clinical and research tool for examining EF among different populations with developmental and mental disabilities (See for example: Ozonoff, 1995; Snow, 1998; Gooding et al., 1999; Romine et al., 2004).

### Statistical Analysis

Separate between subjects ANOVA was conducted for analyzing the differences between the two groups of participants regarding the four above-mentioned psychometric scores. Accordingly, the total errors, the perseverative responses, the perseverative errors and the non-perseverative errors scores were compared between both groups using a one way ANOVA.

## Results

The separate ANOVA revealed a significant difference between the Asthmatic group compared to the non-asthmatic one considering the total errors parameter [*F*(1,55) = 39.53, *p* < 0.001, $\eta_{p}^{2}$ = 0.41]. In addition, a significant difference between the two groups was also found for the perseverative responses [*F*(1,55) = 9.82, *p* = 0.003, $\eta_{p}^{2}$ = 0.15], the perseverative errors [*F*(1,55) = 10.87, *p* = 0.002, $\eta_{p}^{2}$ = 0.16], and for the non-perseverative errors, respectively [*F*(1,55) = 24.87, *p* < 0.001, $\eta_{p}^{2}$ = 0.31] (See **Table 1** for means and SDs).

**Table 1:** Means and SDs of the different psychometric parameters within the different groups and the *F*-values of the ANOVA analysis.

Group		Total errors	Perseverative responses	Perseverative errors	Non-perseverative errors
Non-asthmatic	Mean	15.47	8.27	7.73	7.73
	*SD*±	4.83	4.61	3.84	3.38
Asthmatic	Mean	27.78	13.37	12.04	15.74
	*SD*±	9.44	7.48	5.89	8.05
	*F*-value	39.53^∗∗^	9.82^∗∗^	10.87^∗∗^	24.87^∗∗^


*^∗∗^p < 0.01.*

## Discussion

The current results support previous findings about the poorness of EF among asthmatic children (Yuksel et al., 2008; Fryt et al., 2013). It is important to emphasize that within the current study, children with asthma differ in all the WCST psychometric parameters in comparison to the non-asthmatic group. This result indicates a global poor performance in such test which was used to explore the ability of being concentrated and flexible while demonstrating continues cognitive tasks (Grant and Berg, 1948).

However, one main important parameter that is under consideration was the perseverative errors. This parameter is considered to measure the cognitive flexibility. Accordingly, the results revealed that the asthmatic group showed a poor performance regarding this parameter which indicates poor cognitive flexibility in comparison to the non-asthmatic group.

In light of the fact that both groups had general learning difficulties it would be difficult to deduce that poor EF among the asthmatic group was the main reason for their learning difficulties. However, due to the fact the learning skills are contributed by EF (Kehagia et al., 2010; Best et al., 2011), poor EF among the Asthmatic group might be suggested to be associated with their learning difficulties.

In general, learning difficulties and specifically learning disabilities could be affected by poor EF (Kehagia et al., 2010; Best et al., 2011). Learning performance, in general, needs to be mediated by sufficient sustained attentional resources and continuous concentration during the ongoing performance for enabling effective monitoring and intact cognitive flexibility (St Clair-Thompson and Gathercole, 2006). These abilities, as they were assessed by the different parameters of the WCST, were found to be less effective than those of the non-asthmatic group that already had learning difficulties as well. Accordingly, it seems that learning difficulties of asthmatic children might be related to poor EFs and especially poor cognitive flexibility. In general, the relation between poor EFs and poor learning skills was evident among different populations with learning difficulties like ADHD children as an example (Barkley, 1997, 2001). Alongside, previous studies reported that poor EF was found to be correlated with different academic learning disabilities like Dyslexia (Helland and Asbjørnsen, 2000; Reiter et al., 2005) and mathematical learning disorders (Bull and Scerif, 2001; Toll et al., 2011).

Considering the suggested etiological causes of the comorbidity between Asthma and the poor EF, it can be assumed that the existence of asthma, especially during earlier childhood years, where neural connections are being formed, might be considered as a suggested factor that might interferes the intact development of such neural connections. During this period of development, the existence of frequent and severe asthma attacks could lead into decreases in oxygen levels in the blood, and accordingly this could affect directly the efficient supply of oxygen to various parts of the brain, especially the parts where neural connections continue to develop after birth as in the case of the frontal lobes. This case may leads into the situation of hypo-activation of the frontal lobes. The frontal lobes are the brain regions which are associated with EF development and control (Fuster, 2002). Accordingly, hypo-activation of the frontal lobes may leads into deficits in EF. This assumption about hypo-activationof frontal regions in children with asthma requires future research for examining brain activation during performing cognitive tasks that test EF.

In sum, it is very important to take into consideration the comorbidity between asthma and poor EF. Although the basis of such suggested causation was theoretically explained, it is important to investigate the effectiveness of early intervention programs to reduce the potential negative impact of asthma on later academic performance.

## Ethics Statement

This study was carried out in accordance with the recommendations of ‘Sakhnin college research and ethics committee’ with written informed consent from all subjects. All subjects gave written informed consent in accordance with the Declaration of Helsinki. The protocol was approved by the ‘Sakhnin college research and ethics committee’.

## Author Contributions

HT is the corresponding author. He collected the data and performed the statistical analysis in addition to writing the whole manuscript.

## Conflict of Interest Statement

The author declares that the research was conducted in the absence of any commercial or financial relationships that could be construed as a potential conflict of interest.
